# To Restore Breathing

**Published:** 1879-08

**Authors:** 


*To restore Breathing.*—Place the patient on the floor or ground, face down, and one of the arms under the forehead, to let the fluids escape from the mouth, and the tongue fall forward, leaving the entrance into the windpipe free. Assist this operation by wiping out the mouth. If satisfactory breathing commences use the treatment described below to promote warmth. If there be only slight breathing or no breathing, or if the breathing fail, then turn the patient instantly on the side supporting the head, and excite the nostrils with snuff, hartshorn or smelling salts, or tickle the throat with a feather. Rub the chest and face warm, and dash cold water, or cold and hot water alternately on them. If these methods are unsuccessful lose not a moment, but instantly—*to imitate breathing*—replace the patient on the face, raising and supporting the chest well on a folded coat or other article of dress. Turn the body very gently on the side, and then briskly on the face, and back again, repeating these measures cautiously about fifteen times in a minute, occasionally varying the side.

By placing the patient on his chest the air is forced out; when turned on the side the air enters the chest.

On each occasion that the body is replaced on the face make uniform pressure on the back between and below the shoulder blades, removing the pressure before turning the body on the side. This will induce natural breathing, and, if not too late, *life*.

While the above operations are going on dry the hands and feet, gradually strip the body and substitute dry clothing and blankets, but do not interfere with the efforts to restore breathing.

Should these efforts fail, after two to five minutes' trial, place the patient on the back on a flat surface, inclined a little upward from the feet; raise and support the head and shoulders on a small firm cushion or article of dress placed under the shoulder blades. Draw forward the patient's tongue, and keep it projecting beyond the lips, an elastic band over the tongue and under the chin will answer this purpose, or a piece of tape or string tied around them, or by raising the lower jaw the teeth may be made to retain the tongue in that position. Remove all tight clothing from neck and chest.

